# Treatment with the CCR5 antagonist OB-002 reduces lung pathology, but does not prevent disease in a Syrian hamster model of SARS-CoV-2 infection

**DOI:** 10.1371/journal.pone.0316952

**Published:** 2025-02-05

**Authors:** Bryce M. Warner, Robert Vendramelli, Amrit S. Boese, Jonathan Audet, Nikesh Tailor, Courtney Meilleur, Nathan Glowach, Marnie Willman, Thang Truong, Estella Moffat, Kevin Tierney, Beata Kosak, Irfan Dhanidina, Jarret Engstrom, Bozena Korczak, Ian McGowan, Carissa Embury-Hyatt, Darwyn Kobasa

**Affiliations:** 1 Special Pathogens Program, National Microbiology Laboratory, Public Health Agency of Canada, Winnipeg, Manitoba, Canada; 2 Department of Medical Microbiology and Infectious Diseases, University of Manitoba, Winnipeg, Canada; 3 National Centre for Foreign Animal Diseases, Canadian Food Inspection Agency, Winnipeg, Manitoba, Canada; 4 Orion Biotechnology Polska, Krakow, Poland; 5 Orion Biotechnology, Ottawa, Ontario, Canada; University of Verona, ITALY

## Abstract

Since the emergence of SARS-CoV-2 and the COVID-19 pandemic, a wide range of treatment options have been evaluated in preclinical studies and clinical trials, with several being approved for use in humans. Immunomodulatory drugs have shown success in dampening the deleterious inflammatory response seen in severe COVID-19 patients, but there remains an urgent need for development of additional therapeutic options for COVID-19 treatment. A potential drug target is the CCR5-CCL5 axis, and blocking this pathway may protect against severe disease. Here we evaluated whether OB-002, an analog of human CCL5 and a potent antagonist of CCR5, provides therapeutic benefit in SARS-CoV-2 infected Syrian hamsters. Daily treatment with OB-002 altered immune gene transcription in the lungs, and reduced pathology following infection, but did not prevent weight loss or viral replication in the lungs of infected animals, even in combination with the antiviral drug remdesivir. Our data suggest that targeting the CCR5-CCL5 pathway in SARS-CoV-2 infection in hamsters is insufficient to significantly impact disease development in this model.

## Introduction

The coronavirus disease 2019 (COVID-19) pandemic has been ongoing since March of 2020, following the emergence of Severe Acute Respiratory Syndrome Coronavirus 2 (SARS-CoV-2). This unprecedented pandemic has resulted in the deaths of millions of people globally, significant economic impact, and remains a considerable public health crisis more than three years since it began. Remarkably, within a year of the emergence of SARS-CoV-2, highly effective vaccines were developed, and mass vaccination campaigns were undertaken in many countries. It has been estimated that within the first year of their combined use, vaccines against SARS-CoV-2 may have saved as many as 20 million lives [1]. Despite the significance of this achievement, the emergence of novel SARS-CoV-2 variants of concern, vaccine hesitancy, and global inequities regarding vaccine distribution translates to a continual increase in SARS-CoV-2 infections, and there remains a need for effective therapeutic options for treatment of infection and disease. Many such treatment options have been put forward and tested in different settings, including outpatients and in those who are hospitalized or in intensive care, and range from direct-acting antivirals, immunoglobulins, and immunomodulatory drugs that are either broadly active (e.g. steroids) or targeted (e.g. tocilizumab, an anti-IL-6 monoclonal antibody) [2–12]. Some treatment options have shown promise and have been either authorized or given emergency use authorization by the FDA for treatment of SARS-CoV-2 infection, including the antivirals Paxlovid (nirmatrelvir and ritonavir), molnupiravir, and remdesivir, along with several SARS-CoV-2-specific monoclonal antibodies or cocktails [13–17].

The pathology underlying the disease state in those with severe COVID-19 involves an aberrant immune response that follows the initial acute phase of viral replication [18, 19]. This inflammatory phase of the infection causes an altered immune response characterized by a unchecked inflammation, high cytokine levels, immune cell infiltration, lymphopenia, granulocyte and monocyte abnormalities, and increased IgG production [18, 19]. These perturbations lead to an unregulated inflammatory response. Thus, since early on in the pandemic following the description of the disease course and the involvement of a disrupted immune response, immunomodulatory drugs have been a key area of clinical investigation. Certain drugs such as tocilizumab, an anti-IL-6 monoclonal antibody, dexamethasone, an anti-inflammatory steroid, and baricitinib, a JAK kinase inhibitor, have all shown efficacy in reducing disease by inhibiting excess and deleterious inflammation [10–12, 20]. Despite their success, along with a wide range of other therapeutic options that have gone through some clinical efficacy studies (e.g. Paxlovid, remdesivir, molnupiravir), there is still a strong need for therapeutic options and immunomodulatory drugs for the treatment of COVID-19 patients. One approach that has been proposed is to use the compound cenicriviroc, which is an antagonist of the chemokine receptors CCR2 and CCR5 [21]. Both of these chemokine receptors and their ligands play a role in the development of acute respiratory distress syndrome (ARDS) in the context of SARS-CoV-2 and other viral infections. OB-002, a CCR5 antagonist that is being investigated clinically in the prevention of HIV infection [22, 23] is also being considered as an COVID-19 therapeutic agent.

OB-002, which is also referred to as 5P12-RANTES, is a human CCR5 antagonist that consists of a 69-residue analog of the human protein RANTES/CCL5 [22]. Clinical grade OB-002 can be produced in *Pichia pastoris*, a yeast that is often utilized for highly efficient protein or peptide expression and therefore, can be readily mass produced [24, 25]. OB-002 is a potent antagonist of CCR5 *in vivo* and a topical formulation of OB-002 has shown an excellent safety profile in a Phase 1 clinical trial for HIV prevention [22, 23]. In HIV infection, CCR5 is used by the virus as a co-receptor for cellular entry and OB-002 prevents this entry by inhibiting access to CCR5. Experimental evidence shows that OB-002 is stable in rectal lavage fluid [26] and can protect colorectal tissues from HIV infection *in vitro* [27]. Importantly, *in vivo*, when OB-002 was applied pre-exposure, it fully protected Rhesus macaques in a vaginal simian-human immunodeficiency virus challenge model [28]. Antagonization of CCR5 can prevent the migration of immune cells such as T cells, macrophages, and dendritic cells into tissues in response to infection or chemoattraction by CCR5 ligands CCL3, CCL4, CCL3L1, and RANTES/CCL5 [29]. In the context of SARS-CoV-2 infection, the CCR5-RANTES/CCL5 pathway can attract immune cells to the lungs and amplify the inflammation leading to a deleterious inflammatory outcome [30, 31]. Indeed, preliminary evidence of CCR5 blockade via a monoclonal antibody in humans suggested that CCR5 inhibition can reduce plasma cytokine levels (i.e. mitigate cytokine storm), increase CD8 T cell numbers, and reduce viral RNA levels in the plasma [32]. Based on these findings, we hypothesized that a similar blockade of the CCL5-CCR5 axis in Syrian hamsters infected with SARS-CoV-2 might mitigate disease development in this model.

Here, we investigated whether treatment of SARS-CoV-2 infected hamsters with OB-002 could prevent disease development or reduce pathology. We show that OB-002 treatment did not significantly reduce weight loss or viral replication after infection, though it did alter histopathological scores. Our study suggests that daily OB-002 antagonism of CCR5 during SARS-CoV-2 infection may slightly reduce pathology seen in the lungs of infected animals, but did not affect disease development or viral replication, even in combination with the direct-acting antiviral remdesivir.

## Materials and methods

### Ethics statement

The experiments described were carried out at the National Microbiology Laboratory (NML) of the Public Health Agency of Canada. All experiments were approved by the Animal Care Committee at the Canadian Science Center for Human and Animal Health per the guidelines provided by the Canadian Council on Animal Care, under animal use document # H-20-027. All procedures were performed under inhalation anesthesia using isoflurane with all efforts made to minimize animal suffering and to reduce the number of animals used. All infectious work was performed under biosafety level 4 (BSL-4) conditions. Animals were given food and water *ad libitum* throughout all experiments.

### Cells and viruses

Vero cells (ATCC, Manassas, VA, USA) were cultured in Minimal Essential Medium (MEM) (Hyclone, Logan, UT, USA) supplemented with 5% Bovine Growth Serum (Hyclone, Logan, UT, USA) and L-glutamine. Cells were cultured at 37°C with 5% CO2. SARS-CoV-2 (Canada/ON-VIDO-01/2020; EPI_ISL_425177) was isolated from a positive patient sample and stocks of the virus were grown in VeroE6 cells. Virus stocks were titered by TCID_50_ assay before being used for subsequent *in vivo* experiments. All virus used for *in vivo* experiments was from passage 2 as described previously [33].

### Animal infections and treatments

For infection experiments, groups of 80–100 gram male and female Syrian Golden Hamsters (*Mesocricetus auratus)* were used for all experiments. Hamsters were anesthetized and exposed to 1 x 10^5^ TCID_50_ SARS-CoV-2, by an intranasal route of infection with the inoculum distributed evenly into both nares in a total volume of 100 μL in DMEM diluent. Animals were then monitored daily for clinical signs of disease including lethargy, hunched posture, inactivity, and laboured breathing. Animals were weighed daily until the conclusion of the experiment. Animals that were euthanized at pre-determined time points were deeply anesthetized by inhalation of isoflurane and euthanized by exsanguination via cardiac puncture followed by cervical dislocation. To assess the viral burden in the lung tissues, groups of eight animals were euthanized on days 4 or 7 post-infection as indicated with the remaining eight animals in the first study kept until day 14 to examine weight loss and disease. In the second study, the experiment was terminated on day 7. For the weight loss graphs, all animals from each group are included, regardless of the day they were euthanized during the experiment.

For treatments, animals were anesthetized and administered 67.6 mg/kg of OB-002 prepared in normal saline via intraperitoneal (IP) injection in 100 μL volume. Control (vehicle) animals received the same injection of 100 μL of normal saline. In the first experiment to evaluate OB-002, animals that were infected with SARS-CoV-2 received treatments (either OB-002 or vehicle control) daily for 14 days, until the experiment was ended.

A second experiment was performed to assess the impact of co-treatment of OB-002 for 7 days (on day of infection up to Day 6 post-infection) together with remdesivir treatment on selected days after infection. To prepare remdesivir (MedChemExpress, Monmouth Junction, NJ, USA) for injection it was dissolved in dimethyl sulfoxide to 100 mg/ml and further diluted 2 mg/ml using 12% sulfobutylether-β-cyclodextrin (SBE-β-CD; MedChemExpress, Monmouth Junction, NJ, USA) before injection. Animals treated with only OB-002 and vehicle control animals received the same dose of 67.6 mg/kg as for the first experiment, while the remdesivir only group received an IP injection of 15 mg/kg of the drug in 12% SBE-β-CD in 100 μL on Days 2 and 3 post-infection. The combination remdesivir/OB-002 group received both regimens. The experiment was ended on Day 7 after infection.

### Measurement of viral burden in the tissues

For the measurement of viral titers in the lung tissues of infected animals, TCID_50_ assays were performed. Following necropsy, tissue samples were frozen at -80°C for storage. For infectious assays, lung samples were thawed and placed in 1 mL MEM, supplemented with 1x L-glutamine and 1% FBS, and were homogenized with five mm stainless steel beads in a Bead Ruptor Elite Tissue Homogenizer (Omni International Inc., Keenesaw, GA, USA) at 4 m/s for 30 seconds. Homogenates were clarified by centrifugation at 1500 x g for 10 minutes and ten-fold serial dilutions of tissue homogenates were made in MEM. Dilutions were added to 90–100% confluent Vero cells in triplicate wells and the cytopathic effect was read five days post-infection. TCID_50_ values per gram of tissue were calculated using the Reed and Muench method [34].

For quantification of viral RNA in the lungs, tissue samples harvested for vRNA detection were immersed in RNAlater (Invitrogen, Waltham, MA, USA) at 4°C for 1 day, then stored at −80°C until later use. Tissue samples were thawed, RNAlater removed and then weighed and homogenized in 600 μl RLT buffer using a Bead Ruptor Elite Bead Mill Homogenizer (Omni International Inc., Keenesaw, GA, USA) with a stainless steel bead at 4 m/s for 30 seconds. Viral RNA from 30 mg tissue samples was extracted with the RNeasy Plus Mini kit according to the manufacturer’s instructions (Qiagen, Hilden, Germany). For detection of SARS-CoV-2 RNA, the SARS-CoV-2 E Sarbeco real-time RT–PCR assay, recommended by the WHO was used [33]. The primers used were E_Sarbeco_F1 (5′- ACAGGTACGTTAATAGTTAATAGCGT-3′) and E_Sarbeco_R2 (5′- 554 ATATTGCAGCAGTACGCACACA-3′). The probe was E_Sarbeco_P1 (5′-FAM555 ACACTAGCC/ZEN/ATCCTTACTGCGCTTCG-IowaBlack-3′), primers and probes were purchased from Integrated DNA Technologies (Coralville, IA, USA). The assay was set up using the TaqPath 1-Step Multiplex Master Mix kit (Applied Biosystems, Waltham, MA, USA) on a QuantStudio 5 real-time PCR system (Applied Biosystems, Waltham, MA, USA), as per the manufacturer’s instructions. A standard curve was generated by using plasmids coding for the SARS-CoV-2 E gene and was used on each plate for the quantification of viral copy numbers. Viral copy number per gram of tissue was calculated and shown.

### Histopathology

Tissues were fixed in 10% neutral phosphate buffered formalin for a minimum of 7 days. Tissue samples were embedded and sectioned at 5 μM. A set of slides was stained with hematoxylin and eosin for routine histopathologic examination. Lung lesions were scored out of a total of 15 based on percentage of lung section affected, severity of interstitial pneumonia, and presence/absence of specific histologic lesions. For each animal, a lung section was scored for percentage of lung tissue affected: 0 No pathological changes, 1 <10% of lung section affected, 2 >10% and <50% affected, 3 >50% and <75% affected. Also, each lung section was assigned a score for interstitial pneumonia as follows: 0 not present, 1 mild, 2 moderate, 3 severe. In addition, for each of the following microscopic lesions a score of 0 for not present and 1 for present was assigned: bronchiolitis (including inter-epithelial inflammatory cells, necrosis of bronchiolar epithelium and debris in lumen), diffuse alveolar damage (including necrosis of alveolar epithelial cells, cellular debris in alveoli and intra-alveolar fibrin), alveolar edema, alveolar hemorrhage, vasculitis/endothelialitis, hyperplasia of type II pneumocytes, perivasculitis and presence of multinucleated cells or atypical cells. The board-certified pathologist reviewing the slides was blinded to treatment group.

### Transcriptional profiling of host responses

Tissue RNA was extracted as described above using an RNeasy mini plus kit, which includes a genomic DNA eliminator step. Host mRNA levels of various genes including interleukin-six (IL-6), tumour necrosis factor (TNF), interferon-alpha (IFN-α), interferon-gamma (IFN-γ), RANTES/CCL5, Macrophage inflammatory protein-1 alpha (MIP-1α), interleukin-ten (IL-10), interleukin-1 beta (IL-1β), interleukin-four (IL-4), interleukin-two (IL-2), Interferon-induced GTP-binding protein MX2/MXB (Mx2), Forkhead BoxP3 (FoxP3), Signal transducer and activator of transcription 2 (STAT2), and interferon gamma-induced protein 10 (IP-10) were quantified as described previously using RPL18 as an internal reference gene [35]. RT-qPCR was performed using a TaqPath 1-step RT-qPCR kit as described above for all genes except for IFN-α, MIP-1α, and RANTES/CCL5. Quantification of mRNA transcripts for IFN-α, MIP-1α, and RANTES/CCL5 was done via a two-step RT-qPCR protocol due to the lack of a suitable probe. Briefly, cDNA was made using a SuperScript IV VILO Master Mix kit (Thermo Fisher Scientific, Waltham, MA, USA) according to the manufacturer’s instructions. cDNA from the lungs of each animal was then used for qPCR amplification and detection using SYBR Green MasterMix (Qiagen, Waltham, MA, USA) according to the manufacturer’s instructions. Cycling for the synthesis of cDNA consisted of DNA digestion at 37°C for two minutes followed by primer annealing at 25°C for 10 minutes, reverse transcription at 50°C for 10 minutes, and inactivation of reverse transcriptase at 85°C for five minutes. qPCR using SYBR Green detection was as follows: 10-minute enzyme activation at 95°C followed by 40 cycles of 15 seconds at 95°C and 1 minute at 60°C. Primer and probe sequences for each of the genes detected are included in S1 Table. The Delta delta Ct method was used for calculating fold change in gene expression [35].

### Detection of CCL5 in the serum by ELISA

Detection of hamster CCL5 in the serum of infected animals was done using Nori Hamster RANTES/CCL5 ELISA Kit (Genorise Scientific, Berwyn, PA, USA) according to the manufacturer’s instructions. Serum RANTES/CCL5 concentrations were calculated by comparing unknown serum samples to a standard curve of purified hamster RANTES/CCL5 and interpolation of a standard curve. Quantities are presented as pg/mL of serum.

### Statistical analysis

**Fig 1:**
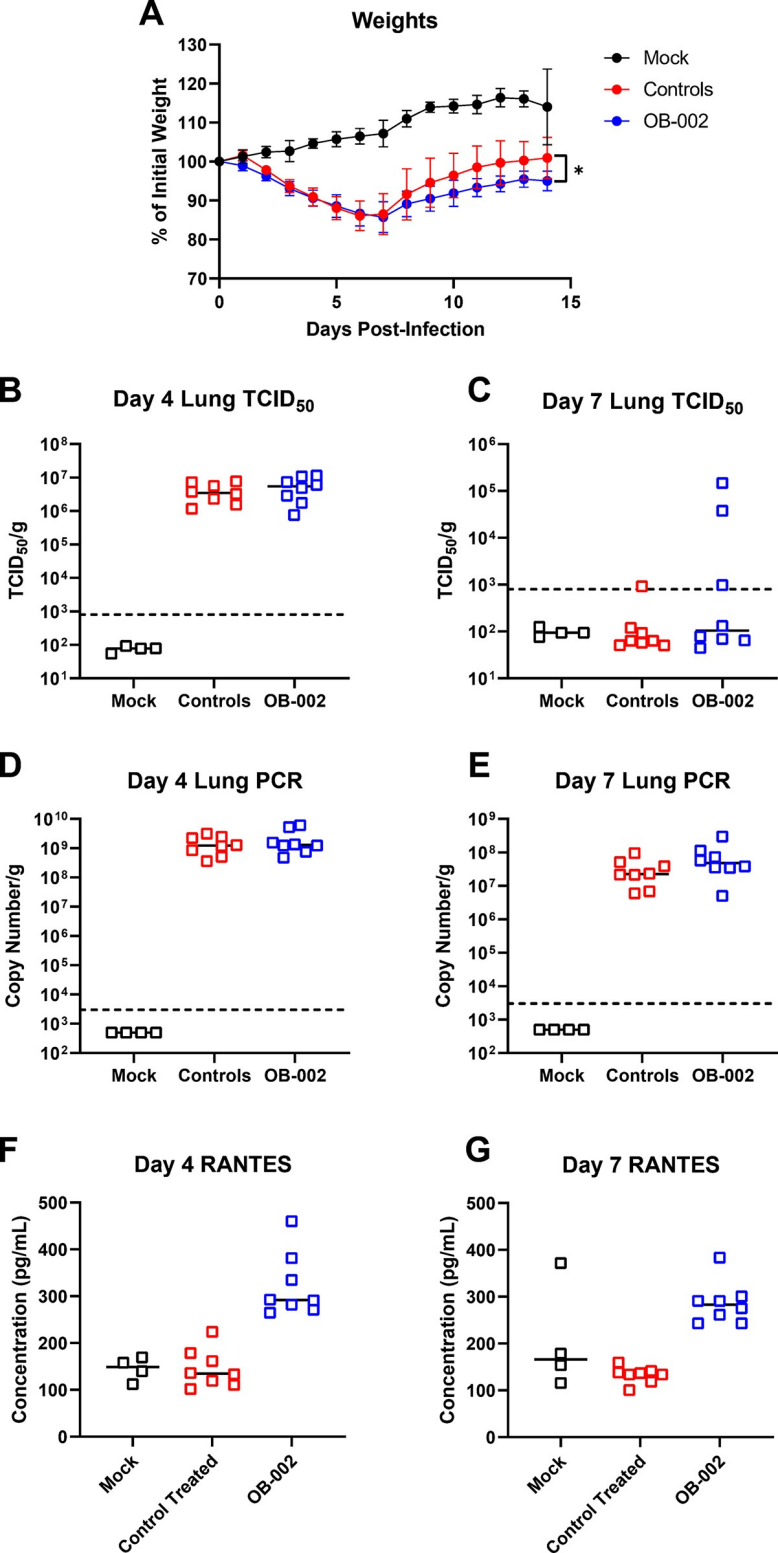
Weight loss and viral replication in SARS-CoV-2 infected, OB-002 treated hamsters. A) Weight loss in SARS-CoV-2 infected hamsters treated with OB-002 or vehicle. Mock, uninfected hamsters are shown for comparison. (n = 24; 16 from Days 5–7, 8 from Days 8–14; and mock n = 12; 8 from Days 5–7, 4 from Days 8–14). B and C) Infectious viral titers in the lungs of animals euthanized on Day 4 (B) or Day 7 (C) post-infection are shown. D and E) Viral RNA levels in the lungs of infected hamsters euthanized on Day 4 (D) or Day 7 (E) are shown. (F and G) Serum concentrations of RANTES/CCL5 as detected by ELISA are shown in each animal euthanized on their respective day post-infection. Shown are means + SD in A, medians in B-G. N = 8 for all TCID_50_, RT-qPCR, or serum ELISA data at each time point, except mock animals which included only 4 animals. In A, Significance was assessed by a mixed effects model with multiple comparisons. * = p<0.05.

Results were analyzed and graphed using Prism 9 software (GraphPad Software, Inc., Boston, MA, USA). For comparison of cytokine gene mRNA expression, comparisons were done using two-tailed Mann-Whitney tests. A mixed effects model with multiple comparisons was used to determine significant differences in weight loss as indicated in Figs 1 and 4.

## Results

### Treatment with OB-002 does not influence disease or infection outcome following SARS-CoV-2 infection in hamsters

To determine whether CCR5 antagonism by OB-002 leads to reduced disease and/or pathology during SARS-CoV-2 infection, we utilized the commonly used Syrian hamster model of infection. We infected groups of hamsters (equal male and female animals per group) with SARS-CoV-2 and then treated the animals daily with OB-002 (67.6 mg/kg/day in 100 μL, n = 24, 8 per timepoint) or the equivalent volume of normal saline as a control (vehicle) (n = 24). A group of mock hamsters was also included for the initial efficacy experiments and for comparison of up- and down-regulation of immune genes (n = 12, 4 per timepoint). All SARS-CoV-2 infected animals lost significant weight (from 4.3 to 21.7%) compared to uninfected controls, with the vehicle and OB-002 treated animals losing comparable weight, peaking on Days 6–7 post-infection (Fig 1A). Weight loss has typically been used as a surrogate for clinical disease in this model and is a relevant measurable for assessing the efficacy of treatment regimens [36]. Both groups began to gain weight by Day 8 post-infection; however, the OB-002 group did not reach their initial mean weights by the end of the study period, while the vehicle controls did. There was a statistically significant difference in mean weight between the OB-002 and vehicle controls at day 15, suggesting a possible detrimental impact of OB-002 treatment on weight gain following infection (Fig 1A). Regardless, OB-002 treatment did not affect weight loss during acute SARS-CoV-2 infection in hamsters. Groups of eight hamsters (four males and four females) were euthanized on Days 4 and 7 following infection to examine viral RNA levels, infectious titers, and lung pathology. On Day 4 post-infection, no infectious titers or viral RNA were detected in mock animals, as expected. Both vehicle and OB-002 groups had high levels of infectious virus in the lungs (5.88 to 7.06 Log_10_ TCID50/g), along with high levels of viral RNA (8.55 to 9.78 Log_10_ E gene copies/g) indicating that OB-002 treatment and CCR5 antagonism does not influence viral clearance (Fig 1B and 1D). On Day 7, infectious titers were significantly reduced compared with those seen on Day 4, and infectious virus was only detectable in a single vehicle-treated animal and three OB-002 animals (Fig 1C). Interestingly, viral RNA levels remained high (6.70 to 8.47 Log_10_ E gene copies/g) on Day 7 in the lungs despite the absence of infectious virus in most cases, possibly due to the accumulation of viral RNA fragments during the acute phase of infection, such as subgenomic mRNA, that are detectable in the PCR assay (Fig 1E).

In our evaluation of the model we examined levels of circulating RANTES/CCL5 in the serum by ELISA. Results showed that OB-002 treated animals had high levels of circulating RANTES/CCL5 (Day 4–322.3 ± 23.9 pg/mL; Day 7–286.2 ± 15.9 pg/mL) in the serum compared with mock (Day 4–144.9 ± 12.5 pg/mL; Day 7–205 ± 57.16 pg/mL) and vehicle-treated controls (Day 4–145.7 ± 14.4 pg/mL; Day 7–132.8 ± 6.08 pg/mL) (Fig 1F and 1G). However, it was determined that the ELISA antigen used to titer circulating RANTES/CCL5 cross-reacts with OB-002, and the RANTES/CCL5 detected in the ELISA was likely circulating drug following daily treatment. These data indicate that infection with SARS-CoV-2 does not induce high levels of expression of RANTES/CCL5 detected as systemic circulation of the protein, since we did not see an increase in circulating CCL5 in vehicle-treated controls. Overall, daily OB-002 treatment of hamsters results in detectable levels of circulating OB-002 which does not influence weight loss or viral replication during SARS-CoV-2 infection in Syrian hamsters.

**Fig 2:**
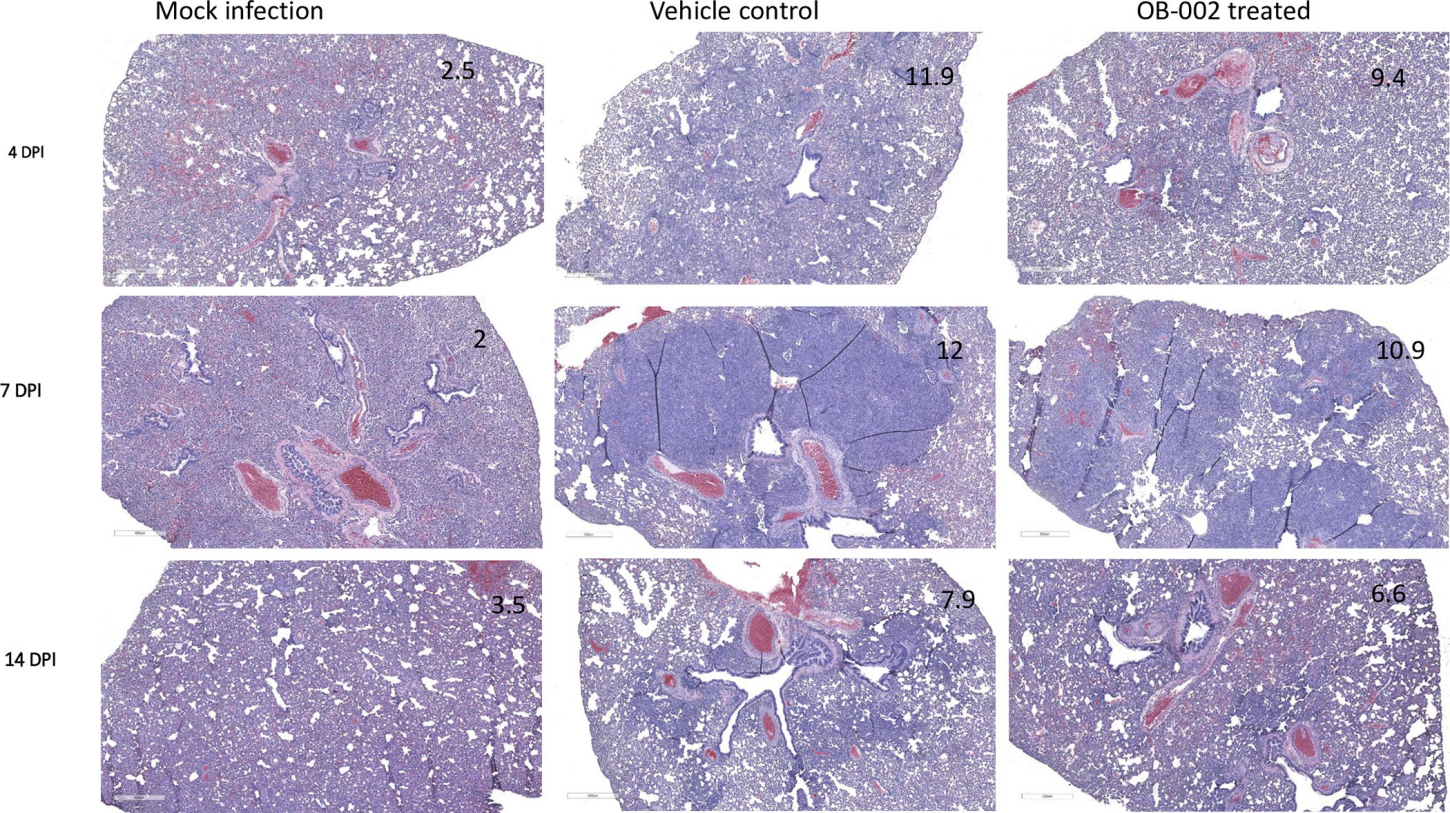
Histopathology seen in SARS-CoV-2 infected, OB-002 treated hamsters. Representative hematoxylin and eosin stained lung samples from hamsters in each group (Mock, vehicle, and OB-002). Histopathological lesion scores for each of the sections are shown in the corner of each image.

We examined the lung tissues collected during infection for histopathological lesions. Lung sections from all animals were examined for microscopic lesions as shown in Fig 2, and were assigned lesion scores. The greatest difference between the vehicle and OB-002 groups was observed on Day 4 post-infection. In the OB-002 treated group, the percentage of lung section affected as well as the number of specific histologic lesions present was decreased leading to a significantly lower total lung lesion score (an average of 9.4 in the OB-002 vs 11.9 in the vehicle) compared to the vehicle control group. There was no significant difference between OB-002 treated or vehicle control group in lung lesion scores on Day 7 and 14 post-infection. Lesions were not observed in mock infected animals; however, there were few areas of interstitial infiltration of inflammatory cells, which is considered normal background.

### Treatment with OB-002 has an immunomodulatory effect in hamsters

**Fig 3:**
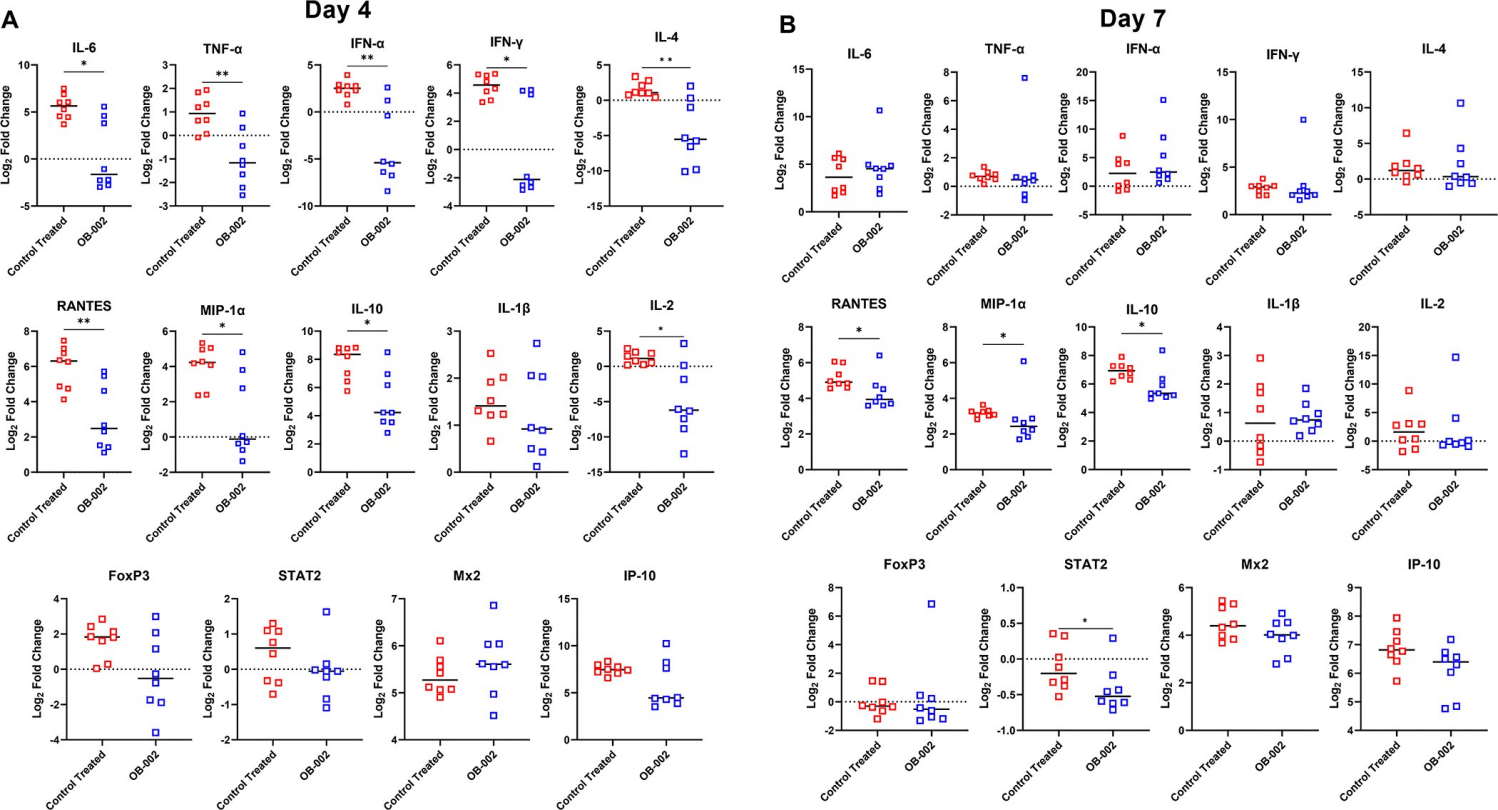
Cytokine and chemokine transcript levels in the lung following infection and OB-002 treatment. Log_2_ fold change in gene transcript levels in OB-002 and vehicle treated hamsters compared with mock hamsters on Day 4 (A) or Day 7 (B) post-infection. Shown are group medians. N = 8 for both groups at both time points. Significance assessed by Mann-Whitney test. * = p <0.05; ** = p <0.01.

Because OB-002 could have immunomodulatory properties as a CCR5 antagonist, we wanted to determine whether treatment impacted the expression of certain cytokines and chemokines in the lungs of the hamsters during infection, even if this did not influence the outcome. Using a previously established set of primer/probe sequences, we compared gene expression levels in lung tissue collected on Days 4 and 7 in the vehicle and OB-002 treated animals compared with mock, uninfected controls (using the delta-delta Ct method) [35]. RANTES/CCL5 mRNA detected in the lungs of infected hamsters, and this was significantly higher in vehicle controls compared with the OB-002 group on both Days 4 and 7 (Fig 3A and 3B). Overall, gene expression was more variable in the OB-002 treated animals, with a few outliers within that group. However, in addition to RANTES/CCL5, there was an overall trend toward reduced transcript levels in the OB-002 group compared to both the vehicle controls, and with respect to mock animals. On Day 4, we detected higher levels of mRNA in the vehicle group for IL-6, TNF-α, MIP-1α, IFN-α, IFN-γ, IL-10, IL-4, and IL-2, while there were no cytokines or chemokines elevated in the OB-002 group (Fig 3A). By Day 7, transcript levels for some genes remained high, but there were fewer differences between the OB-002 and vehicle control groups. RANTES/CCL5, MIP-1α, IL-10, and STAT2 transcript levels were higher on Day 7 in the vehicle controls (Fig 3B). Although some genes demonstrated a high degree of variability, and even clustered trends, within the OB-002 treated animals this did not appear to be due to sex, as differences were randomly distributed between male and female animals. Overall, our data show that while OB-002 treatment did not impact disease development or help facilitate viral clearance, it did significantly influence the transcription level of several important cytokine and chemokine genes in the lungs of infected animals, and thus has some immunomodulatory effect in hamsters. This effect appeared to mostly downregulate transcription, as all impacted genes had lower transcript levels than the vehicle control group.

### Combined treatment with OB-002 and remdesivir does not influence infection outcome

**Fig 4:**
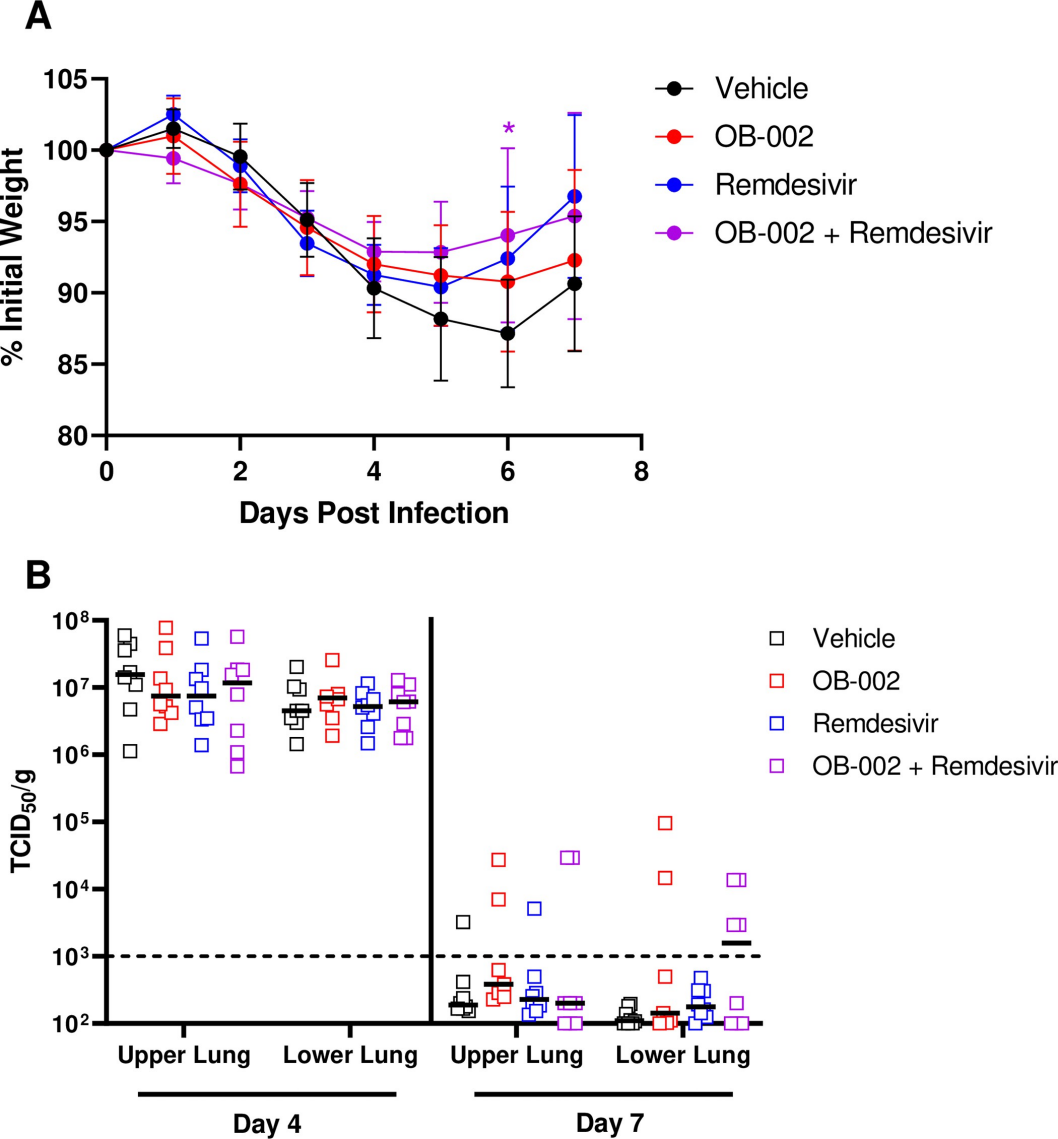
Weight loss and viral replication in SARS-CoV-2 infected, OB-002, and remdesivir treated hamsters. A) Weight loss in SARS-CoV-2 infected hamsters treated with either vehicle, OB-002, remdesivir, or both OB-002 and remdesivir. (n = 16; 8 from Days 5–7). B) Infectious viral titers in the upper and lower lungs of hamsters euthanized on Days 4 or 7 post-infection in each of the treated groups. Shown are mean + SD in A, medians in B. N = 8 for all groups on both days. In A, significance was assessed by a mixed model with multiple comparisons. * = p < 0.05.

Our initial results showed that OB-002 treatment, though it did not have a positive impact on weight loss or viral clearance following SARS-CoV-2 infection, was able to significantly alter the transcription of some cytokines and chemokines that may play a role in disease development in hamsters. To further assess whether these transcriptional changes may influence disease in the presence of a direct-acting antiviral, we performed a second study in which hamsters were treated with normal saline as a vehicle control as before, OB-002 as before, or the antiviral drug remdesivir, or OB-002 and remdesivir in a synergistic combined treatment regimen. Remdesivir is an adenosine nucleoside analog that was developed initially for the treatment of Ebolavirus disease, but has shown antiviral activity against certain viruses, including SARS-CoV-2, and has been approved for use in humans for SARS-CoV-2 infection, despite inconclusive results with respect to its effectiveness. Here, groups of Syrian hamsters were infected and treated with OB-002 or vehicle as above, and the groups being administered remdesivir were given 15 mg/kg on Days 2 and 3 post-infection. In this experiment to evaluate synergy between remdesivir and OB-002, we selected remdesivir treatment on days 2 and 3 post-infection to model treatment after the onset of disease, as this would be more representative of the use of remdesivir in a clinical setting. We monitored and weighed all animals daily, and once again euthanized groups of eight animals on Days 4 and 7 to examine viral loads in the lungs. Compared with vehicle-treated animals, there was only a significant difference in weight loss in the combined OB-002 + Remdesivir group on Day 6 post-infection (Fig 4A). This difference was only seen on Day 6, with animals in all four groups seeing an increase in weight on the final day of the experiment, Day 7. We collected both cranial (upper) and caudal (lower) lung samples to assay for infectious virus and once again, on Day 4, there were high levels of infectious virus in both upper and lower lungs, but no differences between the four groups (Fig 4B). On Day 7, the majority of lung samples did not have detectable infectious virus, however, there were some positive samples, with no discernible or significant differences between the groups. Overall, our data suggests that even in combination with a direct-acting antiviral treatment, OB-002 does not protect against disease nor prevents viral replication following SARS-CoV-2 infection in Syrian hamsters.

## Discussion

Despite the current use of immunomodulatory drugs for the treatment of COVID-19, additional treatment options are needed. There is evidence that targeting the CCR5-CCL5 axis in SARS-CoV-2 infection can lead to a reduction in the immunopathology caused by excessive inflammation [21, 29, 32]. Here we targeted CCR5 directly using OB-002, a peptide produced in yeast that is an analog of human RANTES/CCL5, in hamsters infected with SARS-CoV-2. Daily treatment of hamsters with OB-002 had a significant effect on the expression of several immune genes in the lungs, downregulating transcription compared with vehicle control animals, and was able to reduce the microscopic pathology seen in infected animals. Despite these effects, treatment with OB-002 did not affect weight loss or viral replication when administered alone or in combination with remdesivir, an antiviral nucleoside analog.

Targeting of the CCR5-CCL5 interaction can be achieved through multiple means, and has been widely explored in both HIV prevention [37–39] and treatment strategies, where CCR5 is a co-receptor for viral entry [40, 41]. In the context of COVID-19, the CCR5-CCL5 axis may play a role in the immunopathogenesis of severe cases, and there is theoretical evidence that targeting this pathway could be advantageous in the context of respiratory viral infection. The use of cenicriviroc, a dual CCR2 and CCR5 antagonist, has shown antiviral efficacy *in vitro* against SARS-CoV-2, although in the same study the CCR5-specific inhibitor maraviroc did not show any antiviral effect [42]. A phase 2 trial (NCT04500418) assessing the efficacy of cenicriviroc given to COVID-19 patients has been undertaken, with the hypothesis that treatment will reduce COVID-19 severity through reduction of hyperinflammation [43]. Clinical trials for maraviroc (NCT04435522, NCT04710199) against COVID-19 have also been undertaken [44, 45]. A previous study has shown that blockade of CCR5 using a CCR5-specific antibody leronlimab in human patients can reduce inflammatory cytokines in circulation, impact CD4/CD8 T cell ratios by increasing the number of CD8 T cells, and reduce viral RNA loads in the context of SARS-CoV-2 infection [32]. Multiple phase 2 clinical trials (NCT04343651, NCT04347239, NCT04678830) are being conducted for leronlimab treatment for COVID-19 [45, 46] including for Long COVID [47, 48]. CCR5-CCL5 inhibition has a wide range of effects on the immune response and immune cells, including preventing chemotaxis of CCR5+ macrophages and T cells, prevention of Treg migration into tumours, and blocking CD8 T cell mediated neuropathologies [32, 49, 50].

OB-002 is a more potent inhibitor of the CCR5-CCL5 interaction and has shown to be protective in SHIV infection models in NHPs [28]. Here, though treatment was able to induce changes in immune gene transcript levels, indicating some physiological effects, this was not strong enough to prevent disease in the SARS-CoV-2 hamster model. There may be multiple reasons for this failure. The drug’s half-life and presence in the lungs and respiratory tract may not have been sufficient to induce a strong enough blockade of CCR5 to prevent infiltration of immune cells and excess inflammation, even though several immune genes were downregulated in the lungs. The genes that were downregulated in the lungs following OB-002 treatment include mostly innate and adaptive pro-inflammatory cytokines, with the exception of RANTES and IL-10. A reduction in these cytokines would in theory reduce immune infiltration and immune-mediated pathology, however we examined these reductions on days 4 and 7 post-infection. We may have measured sustained inflammatory responses in vehicle treated animals, while earlier inflammatory responses may not have been as affected by OB-002 treatment. Our ELISA data indicate that OB-002 was likely present in the serum at high levels after administration, thereby tangentially indicating that CCR5 is not a critical player in the development of disease in hamsters infected with SARS-CoV-2. Because OB-002’s effect only acts on the immune system and not directly against some stage of the viral replication cycle, the impacted immune target(s) would need to play a significant role in disease development. Additionally, the disease seen in hamsters is mild compared to severe disease that can occur in humans, leading to hospitalization, ICU admittance, and sometimes death. It is possible that the hyperinflammation seen in humans during severe disease does not occur in hamsters to a degree where CCR5 blockade would have a measurable or noticeable effect. Regardless, in the hamster model, CCR5 antagonism does not impact disease development or viral replication.

Since we observed changes in immune gene transcript levels and microscopic pathology with OB-002 treatment, we hypothesized that OB-002 given in combination with a direct-acting antiviral may have a synergistic protective effect in SARS-CoV-2 infected hamsters. Therefore, we repeated the original study and included groups treated either with remdesivir alone or remdesivir with OB-002. The only measurable change noted was a small but significant reduction in weight loss in the combination group on Day 6 post-infection, but not on any other days. Overall, no treatment regimens protected hamsters from weight loss or viral replication in the lungs in this experiment, similar to the first study. One limitation to this experiment is that remdesivir is quickly metabolized in hamsters, reducing the amount of the active form of the drug to concentrations that may be too low to have a strong antiviral effect, although treatments of viral infections have been reported in hamsters and mice with remdesivir [51, 52]. Treatment with remdesivir alone has been shown to reduce weight loss and viral lung titers in SARS-CoV-2 infected hamsters when administered on the day of infection [53]. Indeed remdesivir and its prodrug form have shown efficacy in mouse and ferret SARS-CoV-2 infection models [51, 52, 54]. A form of the prodrug that is more bioavailable may be a more suitable option for studies moving forward to see if immune modulation with OB-002 can work in concert with antiviral therapies. In addition, OB-002’s use with other, more efficacious antivirals such as molnupiravir or Paxlovid, may better illustrate its synergistic capabilities, as there have been mixed results reported on the therapeutic effectiveness of remdesivir for SARS-CoV-2 [55, 56].

We sought to determine whether CCR5 antagonism via OB-002 administration in hamsters would be beneficial during SARS-CoV-2 infection. Here we reported negative experimental data showing that treatment did not significantly alter disease course or reduce viral replication in the lungs, despite having some physiological effect in terms of changing gene transcription. Our data suggests that targeting the CCR5-CCL5 axis in the context of SARS-CoV-2 infection does not significantly impact disease development in the hamster model, even in the presence of direct antiviral treatment. Despite the use of other CCR5 blocking therapies such as leronlimab previously showing some efficacy in humans, the FDA does not currently support its use, and it is apparent that targeting of this specific immune axis is insufficient in preventing COVID-19. Alternative immunomodulatory drug options such as anti-IL-6 monoclonal antibody treatment or steroid use appear to be preferable options. Ultimately, there should be a continued search for alternative immune targets in SARS-CoV-2 infection.

## Supporting information

S1 Table Primer and probe sequences for hamster immune genes. All probe sequences include a 5`6-FAM (Fluorescein) fluorescent dye and a 3`IBFQ (Iowa Black) quencher.
(DOCX)
