# Supplementary material for: Treatment with the CCR5 antagonist OB-002 reduces lung pathology, but does not prevent disease in a Syrian hamster model of SARS-CoV-2 infection
Source: PLoS One. 2025 Feb 5;20(2):e0316952. doi: 10.1371/journal.pone.0316952 (PMC11798459; doi:10.1371/journal.pone.0316952)
Supplement: S1 Table — All probe sequences include a 5`6-FAM (Fluorescein) fluorescent dye and a 3`IBFQ (Iowa Black) quencher. (DOCX) [file pone.0316952.s001.docx]

| ***Gene*** | ***Primer/Probe*** | ***Sequence*** |
| --- | --- | --- |
| IL-6 | Forward | CCTGAAAGCACTTGAAGAATTCC |
|  | Reverse | GGTATGCTAAGGCACAGCACACT |
|  | Probe | AGAAGTCACCATGAGGTCTACTCGGCAAAA |
| TNF-α | Forward | GGAGTGGCTGAGCCATCGT |
|  | Reverse | AGCTGGTTGTCTTTGAGAGACATG |
|  | Probe | CCAATGCCCTCCTGGCCAACG |
| IFN-α | Forward | AGACTGGGAGTTGCCTGTGA |
|  | Reverse | GAGGAATCCAGGGCTTTCCAG |
| IFN-γ | Forward | GGCCATCCAGAGGAGCATAG |
|  | Reverse | TTTCTCCATGCTGCTGTTGAA |
|  | Probe | CACCATCAAGGCAGACCTGTTTGCTAACTT |
| RANTES/CCL5 | Forward | TCAGCTTGGTTTGGGAGCAA |
|  | Reverse | TGAAGTGCTGGTTTCTTGGGT |
| MIP-1α | Forward | GGTCCAAGAGTACGTCGCTG |
|  | Reverse | GAGTTGTGGAGGTGGCAAGG |
| IL-10 | Forward | GTTGCCAAACCTTATCAGAAATGA |
|  | Reverse | TTCTGGCCCGTGGTTCTCT |
|  | Probe | CAGTTTTACCTGGTAGAAGTGATGCCCCAGG |
| IL-1β | Forward | GGCTGATGCTCCCATTCG |
|  | Reverse | CACGAGGCATTTCTGTTGTTCA |
|  | Probe | CAGCTGCACTGCAGGCTCCGAG |
| IL-4 | Forward | CCACGGAGAAAGACCTCATCTG |
|  | Reverse | GGGTCACCTCATGTTGGAAATAAA |
|  | Probe | CAGGGCTTCCCAGGTGCTTCGCAAGT |
| IL-2 | Forward | GTGCACCCACTTCAAGCTCTAA |
|  | Reverse | AAGCTCCTGTAAGTCCAGCAGTAAC |
|  | Probe | AGGAAACCCAGCAGCACCTCGAGC |
| Mx2 | Forward | CCAGTAATGTGGACATTGCC |
|  | Reverse | CATCAACGACCTTGTCTTCAGTA |
|  | Probe | TGTCCACCAGATCAGGCTTGGTCA |
| FoxP3 | Forward | AAGCAGATCACCTCCTGGAT |
|  | Reverse | AGCTGCTGCTCCAGAGAC |
|  | Probe | CACCACTTCTCTCTGGAGGAGGCAC |
| IP-10 | Forward | GCCATTCATCCACAGTTGACA |
|  | Reverse | GCCATTCATCCACAGTTGACA |
|  | Probe | CGTCCCGAGCCAGCCAACGA |
| STAT2 | Forward | AATGCCTTCAGAGTGTACCG |
|  | Reverse | TGTTCACCGTACTATCCACTTCAT |
|  | Probe | CTGAAGTCAGGACCGCATACTCAGGA |
| RPL18 | Forward | GTTTATGAGTCGCACTAACCG |
|  | Reverse | TGTTCTCTCGGCCAGGAA |
|  | Probe | TCTGTCCCTGTCCCGGATGATC |
